# 

**Published:** 1917-06

**Authors:** 


### ANNOUNCEMENTS

EXAMINATION OF DENTISTS FOR THE UNITED STATES ARMY. The surgeon-general of the army announces that examinations for the appointment in the Dental Corps of the Army will be held at Fort Slocum, N. Y.; Columbus Barracks, Ohio; Jefferson Barracks, Mo.; Fort Logan, Colo., and Fort McDowell, Cal., on Monday, July 2, 1917.

Application blanks and full information concerning these examinations can be procured by addressing the surgeon-general United States Army, Washington, D. C.

The essential requirements to securing an invitation are that the applicant shall be a citizen of the United States, shall be between twenty-one and thirty-two years of age, a graduate of a dental school legally authorized to confer the degree of D.D.S., and shall be of good moral character and habits.

Successful applicants will be immediately appointed—as far as the number of vacancies will permit—to the grade of first lieutenant, at an entrance salary of two thousand dollars per annum.

In order to perfect all necessary arrangements for the examination, applications must be in the possession of the surgeon-general at least two weeks before the date of examination; early attention is therefore enjoined upon all intending applicants. There are at present fourteen vacancies to be filled, and on July 1, 1917, there will be twenty-two additional vacancies.

---

WORK FOR THE AMERICAN AMBULANCE HOSPITAL IN PARIS. Mrs. Freer, of Chicago, has by her own efforts raised from fairs and other entertainments in Chicago about ten thousand dollars for this great relief work. She is asking dentists of the country to send her gifts for this cause. She would like to be able to send funds every month to the support of this hospital. Can't you give one dollar a month? If so, send it regularly to Mrs. Archibald Freer, 1420 Lake Shore Drive, Chicago.

---

THE NATIONAL DENTAL ASSOCIATION will meet in New York City October 22 to 26, 1917. Extensive preparations are being made for this meeting. It will undoubtedly be a great meeting. Make your plans to attend.

